# Influence of angiographic spontaneous coronary reperfusion on long-term prognosis in patients with ST-segment elevation myocardial infarction

**DOI:** 10.18632/oncotarget.19338

**Published:** 2017-07-18

**Authors:** Xiaoming Li, Boyu Li, Jing Gao, Yunfei Wang, Song Xue, Dachuan Jiang, Qi Hua, Jing Li

**Affiliations:** ^1^ Division of Cardiology, Xuanwu Hospital Capital Medical University, Beijing, China

**Keywords:** ST-segment elevation myocardial infarction, spontaneous reperfusion, coronary angiography, prognosis

## Abstract

**Objective:**

To explore the influence of angiographic spontaneous coronary reperfusion (SR) on the short- and long-term outcomes in patients with ST-segment elevation myocardial infarction (STEMI).

**Materials and Methods:**

Consecutive patients diagnosed as STEMI and undergoing emergent coronary angiography from January 2009 to August 2010 in a single center were enrolled. Patients whose initial coronary blood flow met Thrombolysis in Myocardial Infarction (TIMI) grade 3 were regarded as angiographic SR. Others (TIMI grade < 3) were included into the NSR group and subsequently underwent primary percutaneous coronary intervention (PCI). Patients’ characteristics and outcomes were compared.

**Results:**

A total of 207 patients were eligible for analysis. The coincidence rate of patients both with ≥ 70% ST-segment resolution and ≥ 70% relief of chest pain and SR was 100%. Patients in the SR group (*n* = 38) were younger, had more smokers, with higher level of platelet count, lower blood glucose and uric acid, and more distal culprit lesions, as compared to the NSR group (*n* = 169). Importantly, patients in the SR group had lower rates of in-hospital events (0 vs. 22.5%, *p* < 0.001) with less stents placed (1.03 ± 1.05 vs. 1.59 ± 1.17, *p* = 0.007). Moreover, there were comparable long-term outcomes (2.6% vs. 6.9%, *p* = 0.302) between the two groups during 41-month follow ups.

**Conclusions:**

Angiographic SR is associated with significantly favorable short-term outcomes.

## INTRODUCTION

Reperfusion strategy, including fibrinolytic therapy or primary percutaneous coronary intervention (PCI), is the key point to reduce mortality in ST-segment elevation myocardial infarction (STEMI). In daily practice, it is not rare that some patients may achieve reopening of the culprit artery without undergoing any reperfusion therapy, which is termed as spontaneous reperfusion (SR).

In previous studies, SR was defined as initial coronary flow of Thrombolysis in Myocardial Infarction (TIMI) [1] grade 3 assessed by emergent angiography [2–4]. Frequency of SR varies around 10%–30% [5–9]. Compared to non-SR (NSR) patients, SR is associated with a lower rate of short-term mortality or heart failure [2–5, 7, 9]. However, the relationship between SR and patient characteristics is still unclear. Moreover, fewer studies have reported the influence of SR on treatment strategy or long-term outcomes.

In the current study, we collected patients diagnosed with STEMI and tried to investigate the relation between clinical features and SR. We also explore the relationship of ST segment resolution and symptom relief and SR. Moreover, in-hospital events and long-term outcomes were compared between SR patients and NSR patients.

## MATERIALS AND METHODS

### Study population

In this retrospective study, STEMI patients, who had chest pain within 12 h and underwent emergency coronary angiography in one medical center from January 2009 to August 2010, were included. Exclusion criteria: 1) patients received thrombolytic therapy; 2) patients had prior myocardial infarction; 3) Patients refused angiography. Patients were divided into SR group and NSR group. SR referred to the culprit vessel achieved coronary blood flow of TIMI grade 3 identified by initial angiogram.

All patients received 300 mg of aspirin loading dose and 300 mg of clopidogrel, followed by 100 mg of aspirin maintenance dose and 75 mg of clopidogrel per day. Patient characteristics and in-hospital events were collected by questionnaires or medical records. In-hospital events referred to composite of any-cause death, reinfarction, congestive heart failure and cardiogenic shock [10]. Reinfarction was identified as an acute myocardial infarction occurred during the time of in the hospital after an incident myocardial infarction. Reinfarction was considered when ST elevation ≥ 0.1 mV recurs, or new pathognomonic *Q* waves appear, in at least two contiguous leads, particularly when associated with ischaemic symptoms for 20 min or longer. If the cTn concentration is elevated, but stable or decreasing at the time of suspected reinfarction, the diagnosis of reinfarction requires a 20% or greater increase of the cTn value in the second sample. If the initial cTn concentration is normal, the criteria for new acute myocardial infarction apply [11]. All biochemistry indexes were tested in the center laboratory within 24 hours.

### Diagnostic criteria for STEMI

If following criteria were present: the presence of continuous chest pain lasting ≥ 20 minutes; and either 1) ST-segment elevation of ≥ 2 mm in at least 2 contiguous precordial leads, or 2) ST-segment elevation of ≥ 1 mm in at least 2 inferior leads, or 3) new left bundle branch block [11]. It was later confirmed by the elevation of myocardial biomarker (CK-MB or troponin) ≥ 2 folds of normal value.

### Chest pain assessment

Chest pain was assessed with a numerical rating scale (NRS). Patients were asked to rate their pain on an 11-point scale, where 0 indicated no pain and 10 indicated the worst imaginable pain [12] at the admission (emergency room or at home). Reduction in pain for each patient was inquired before initiation of emergency coronary angiography. Symptom relief was defined as above 50% easement of pain as compared to the top level of pain for each patient.

### ST segment resolution assessment

Electrocardiograms were examined at the admission and initiation of emergency coronary angiography. ST segment resolution was defined as 50–100% reduction in sum ST elevation.

### Coronary angiography

Coronary angiography was performed using 5–6 French Judkins catheters through femoral or radial approaches [13]. The decision to perform PCI was made based on the discretion of operators. Usually, if patients in the SR group had significant stenosis in the target vessel or other vessels, we preferred to perform an elective PCI after 7 days of the initial angiography.

### Long-term follow up

Follow up information was collected via phone call every 6 months. Primary endpoint was defined as major adverse cardiovascular and cerebrovascular events (MACCE), referring to composite of any-cause death, non-fatal cardiac arrest, acute coronary syndrome, congestive heart failure and stroke [14]. If any event occurred, primary endpoint was reached. This study was approved by the ethics committee of the hospital and the informed consents were obtained from patients. All authors have approved the manuscript and we confirm that all methods were performed in accordance with the relevant guidelines and regulations. The full disclosure of any potential conflict of interest has been declared.

### Statistical analysis

Continuous variables are presented as mean ± SD or if not normally distributed as median with interquartile range. Two-sample *t*-tests or rank-sum tests were used to test group differences. For categorical variables, the data were summarized in percentages and X^2^ or Fisher exact test was used to assess group differences, where appropriate. Multivariate logistic regression analysis was performed to estimate odds ratios (OR) and 95% confidence intervals (95% CI) to identify independent predictors of SR. Long-term survival of MACCE was estimated with the Kaplan-Meier method. The Cox proportional hazard survival model was used to assess the association between SR and long-term outcomes while adjusting for potential confounders. A value of *p* < 0.05 was considered significant. All tests were two-sided, with a 5% level of significance. All analyses were performed using SPSS software 13.0 (SPSS Inc; Chicago, IL, USA).

## RESULTS

### Baseline characteristics

**Figure 1:**
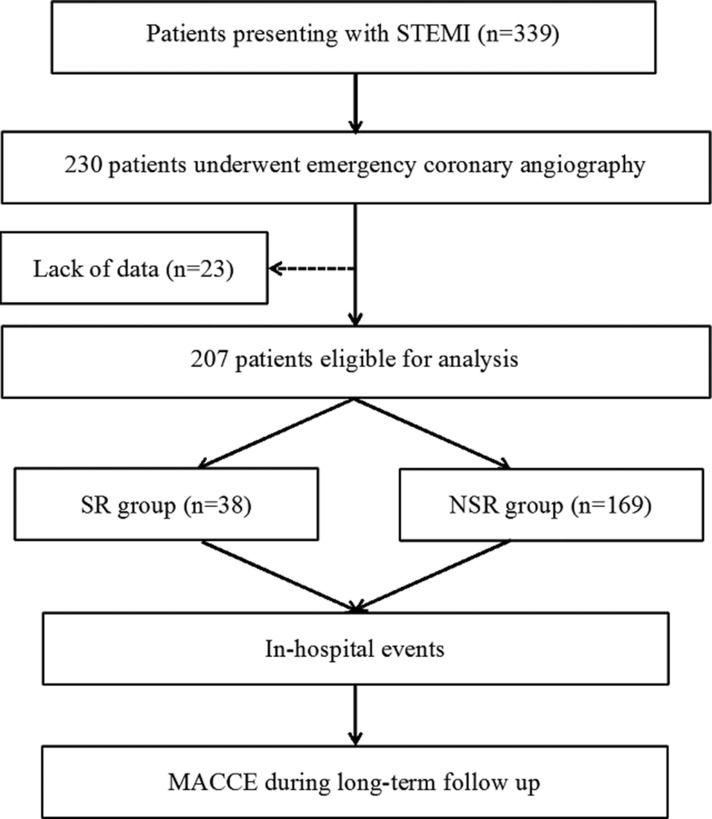
Flow chart of the study MACCE = major and adverse cardiovascular and cerebrovascular events; NSR = non-spontaneous reperfusion; SR = spontaneous reperfusion; STEMI = ST-segment elevation myocardial infarction.

A total of 207 patients were eligible for analysis. Among them, 38 (18.4%) met the criteria of SR. Study flowchart is shown in Figure 1. In the NSR group, 130 (76.9%) patients got a TIMI flow grade 3 after emergent PCI. Patients in the SR group were younger (56.76 ± 12.89 vs. 61.80 ± 11.78, *p* = 0.020) and more smokers (73.7% vs. 53.3%, *p* = 0.022). The platelet (266.64 ± 66.91 × 10^9^/L vs. 234.49 ± 66.86 × 10^9^/L, *p* = 0.010), blood level of uric acid (298.03 ± 74.46 × μmol/L vs. 340.47 ± 106.89 × μmol/L, *p* = 0.025) and glucose (6.04 × mmol/L vs. 7.98 × mmol/L, *p* = 0.005) levels were statistically different between SR and NSR group (Table 1).

**Table 1:** Baseline characteristics

	SR Group	NSR Group	*P*
	(*n* = 38)	(*n* = 169)	
Age (years)	56.76 ± 12.89	61.80 ± 11.78	0.020
Gender (Male/Female)	34/4	131/38	0.098
Hypertension (%)	18 (47.4)	85 (50.3)	0.744
Diabetes mellitus (%)	8 (21.1)	39 (23.1)	0.788
Smoking (%)	28 (73.7)	98 (53.3)	0.022
Anterior wall myocardial infarction (%)	22 (57.9)	69 (40.8)	0.055
Systolic blood pressure (mm Hg) [median (Q1, Q3)]	134 (112–147)	135 (112–146)	0.451
Diastolic blood pressure (mm Hg) [median (Q1, Q3)]	79 (70–86)	78 (66–88)	0.761
Heart rate (/min) [median (Q1, Q3)]	78 (67–86)	75 (63–88)	0.288
Time from symptom onset (h) [median (Q1, Q3)]	4.9 (2.4–6)	4.5 (2.5–5)	0.505
EF (%)	59 ± 9	56 ± 10	0.126
Time from symptom onset to PCI (min) [median (Q1, Q3)]	195 (175–365)	347 (210–395)	0.419
Killip cardiac functional grading I-II (%)	36 (94.7)	162 (95.9)	0.759
GP IIb/IIIa antagonist before procedures (%)	3 (14.3)	9 (8.6)	0.398
Admission drug treatment			
Aspirin (%)	38 (100)	159 (94.1)	0.124
Clopidogrel (%)	38 (100)	158 (93.5)	0.106
Beta-blockers (%)	33 (86.8)	123 (76.8)	0.069
ACE inhibitors (%)	37 (97.4)	146 (86.45)	0.056
Nitrates (%)	36 (94.7)	141 (83.49)	0.267
Low-molecular-weight heparins (%)	38 (100)	167 (98.8)	0.438
Platelet (10^9^/L)	266.64 ± 66.91	234.49 ± 66.86	0.010
Blood glucose (mmol/L)	6.04 (4.30–7.36)	7.98 (5.65–8.77)	< 0.001
Uric acid (μmol/L)	298.03 ± 74.46	340.47 ± 106.89	0.025
High density lipoprotein (mmol/L)	1.04 ± 0.19	1.06 ± 0.24	0.687
Low density lipoprotein (mmol/L)	2.62 ± 0.81	2.62 ± 0.80	0.982
Serum creatinine (mol/L) [median (Q1, Q3)]	69.1 (61.0–77.8)	76.9 (60.0–82.0)	0.507

Data are presented as percentage or median (25th, 75th centile), Q1 = 25th quartiles, Q3 = 75th quartiles, where appropriate. ACE = angiotensin converting enzyme, EF = ejection fraction, GP = Glycoprotein, NSR = non-spontaneous reperfusion, PCI = percutaneous coronary intervention, SR = spontaneous reperfusion.

In the SR group, 22 (57.9%) patients had severe (≥ 90%) stenosis in culprit artery, while 10 (26.3%) patients with stenosis about 80–89%, 2 (5.3%) with stenosis about 70–79% and 4 (10.5%) with < 70% stenosis.

The culprit lesions were more distal in the SR group than those in the NSR group (26.3% vs. 9.5%, *p* < 0.005). Stents were placed in 4 (10.5%) patients of the SR group and 143 (84.6%) of the NSR group during the initial procedure (*p* < 0.001) (Table 2). Number of stents placed in emergent procedure was far less in the SR group than that of the NSR group (0.18 ± 0.61 vs. 1.16 ± 0.70, *p* < 0.001). After emergent and selective PCI, patients in the SR group still had a lower rate of stent placement than the NSR group (60.5% vs. 86.4%, *p* = 0.020) (Table 2). The total number of stents used for treating culprit lesions in the SR group was less than that in the NSR group (1.03 ± 1.05 vs. 1.59 ± 1.17, *p* = 0.007) (Table 2).

**Table 2:** Angiographic information

	SR Group (*n* = 38)	NSR Group (*n* = 169)	*P*
Infarct-related artery			0.024
LAD (%)	21 (55.3)	71 (42.0)	
LCX (%)	9 (23.7)	23 (13.6)	
RCA (%)	8 (21.1)	75 (44.4)	
Infarct-related artery was LAD (%)	21 (55.3)	71 (42.0)	0.233
Distal lesions (%)	10 (26.3)	16 (9.5)	0.005
Vessel disease			0.510
One-vessel disease (%)	18 (47.4)	74 (43.8)	
Two-vessel disease (%)	12 (31.6)	44 (26.0)	
Three-vessel disease (%)	8 (21.1)	51 (30.2)	
Number of stents placed in emergent procedure	0.18 ± 0.61	1.16 ± 0.70	< 0.001
Emergent stent placement	4 (10.5)	143 (84.6)	< 0.001
Total number of stents placed	1.03 ± 1.05	1.59 ± 1.17	0.007
Patients undergoing PCI (%)	23 (60.5)	146 (86.4)	0.020
Stent diameter (mm)	3.44 ± 0.36	3.38 ± 0.45	0.545
Total stent length (mm)	42.3 ± 18.4	45.5 ± 28.0	0.740

LAD = left anterior descending branch, LCX = left circumflex artery, NSR = non-spontaneous reperfusion, RCA = right coronary artery, PCI = percutaneous coronary Intervention, SR = spontaneous reperfusion.

### Clinical predictors for SR

The coincidence rate of patients both with ≥ 70% ST-segment resolution and ≥ 70% relief of chest pain and SR was 100%.

### Factors related to SR

Multiple variable analysis showed platelet (OR, 1.007; 95% CI: 1.001–1.013; *p* = 0.019), blood glucose (OR, 0.750; 95% CI: 0.622–0.905; *p* = 0.003), uric acid (OR, 0.993; 95% CI: 0.800–0.998; *p* = 0.011), and culprit lesion in distal lesions (OR, 0.107; 95% CI: 0.029–0.393; *p* = 0.001) or left anterior descending branch (LAD) (OR, 0.285; 95% CI: 0.105–0.711; *p* = 0.013) were independently associated with SR (Table 3).

**Table 3:** Factors related to spontaneous reperfusion

	B	OR (95% CI)	*P*
Age (years)	−0.012	0.988 (0.945–1.033)	0.592
Gender	0.550	1.733 (0.391–7.682)	0.469
EF (%)	0.010	1.011 (0.600–1.060)	0.677
Infarct-related artery was LAD	−1.256	0.285 (0.105–0.711)	0.013
Distal lesions	−2.239	0.107 (0.029–0.393)	0.001
Platelet (10^9^/L)	0.007	1.007 (1.001–1.013)	0.019
Neutrophils (10^9^/L)	−0.030	0.970 (0.833–1.130)	0.698
Blood glucose (mmol/L)	−0.287	0.750 (0.622–0.905)	0.003
Uric acid (μmol/L)	−0.007	0.993 (0.800–0.998)	0.011
High density lipoprotein (mmol/L)	−0.696	0.499 (0.046–5.459)	0.569
Low density lipoprotein (mmol/L)	0.244	1.277 (0.711–2.291)	0.413
Serum creatinine (mol/L)	0.005	1.001 (0.962–1.062)	0.662

CI = confidence interval, EF = ejection fraction, LAD = left anterior descending branch, OR = Odds ratio.

### In-hospital events

Patients in the SR group had a significantly lower rate of in-hospital events as compared to the NSR group, including total events (0 vs. 22.5%, *p* < 0.001), any-cause death (0 vs. 5.9%), reinfarction (0 vs. 3.0%), congestive heart failure (0 vs. 7.1%) and cardiogenic shock (0 vs. 6.5%). The in-hospital mortality of the SR group had no statistically significant difference compared to those in the NSR group (0% vs. 5.9%, *p* = 0.125).

### Long-term outcome

**Figure 2:**
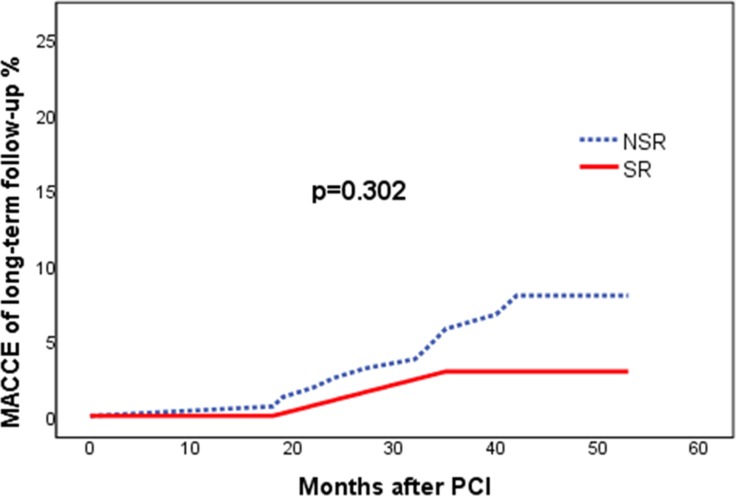
Kaplan-Meier curves for major and adverse cardiovascular and cerebrovascular events MACCE = major and adverse cardiovascular and cerebrovascular events; NSR = non-spontaneous reperfusion; PCI = percutaneous coronary intervention; SR = spontaneous reperfusion.

A total of 197 patients were enrolled in the follow up of the study. Median duration of the follow up was 41 months (Quartile 1: 37 months and Quartile 3: 45 months). The longest follow up was 53 months. There were 16 (8.1%) patients lost follow-up (4 patients in the SR group and 16 patients in the NSR group, 10.5% vs. 7.5%, *p* = 0.546). Patients in the SR group had a non-statistically lower rate of MACCE as compared to the NSR group (2.6% vs. 6.9%, *p* = 0.302) (Figure 2). Age (OR, 1.077; 95% CI: 1.014–1.143; *p* = 0.016) and hypertension (OR, 5.201, 95% CI: 1.134–23.855; *p* = 0.034) were independent predictors of long-term events (Table 4).

**Table 4:** Cox proportional hazards regression of long-term clinical outcomes

	OR (95% CI)	*P*
Age (years)	1.077 (1.014–1.143)	0.016
Gender	1.960 (0.563–6.823)	0.291
Hypertension	5.201 (1.134–23.855)	0.034
Diabetes mellitus	0.714 (0.171–2.977)	0.644
Smoking	1.112 (0.277–4.456)	0.881
SR	0.232 (0.032–1.669)	0.304
Killip cardiac functional grading I-II	0.147 (0.064–0.381)	0.147

CI = confidence interval, OR = Odds ratio, SR = spontaneous reperfusion.

As 23.1% of patients in the NSR group did not achieve blood flow of TIMI grade 3 finally, which may attribute to poor outcomes. We therefore analyzed the data again after excluding patients with slow blood flow. Eventually, patients in the SR (*n* = 38) group still had a lower rate of in-hospital events (0 vs. 16.9%, *p* = 0.007) compared to the NSR (*n* = 130) group and the two groups had the similar long-term outcomes (2.6% vs. 6.3%, *p* = 0.378). Age (OR, 1.095; 95% CI: 1.024–1.171; *p* = 0.008) and hypertension (OR, 5.818, 95% CI: 1.214–27.878; *p* = 0.028) were independent predictors of long-term events (Table 5).

**Table 5:** Cox proportional hazards regression of long-term clinical outcomes after excluding patients with slow blood flow

	OR (95% CI)	*P*
Age (years)	1.095 (1.024–1.171)	0.008
Gender	1.405 (0.335–5.899)	0.642
Hypertension	5.818 (1.214–27.878)	0.028
Diabetes mellitus	0.188 (0.022–1.650)	0.132
Smoking	0.773 (0.188–3.173)	0.721
SR	0.522 (0.062–4.365)	0.548
Killip cardiac functional grading I-II	0.629 (0.173–2.278)	0.480

CI = confidence interval, OR = Odds ratio, SR = spontaneous reperfusion.

## DISCUSSION

In this study, we found the incidence of SR was 18.4%. Combination of ≥ 70% ST-segment resolution with ≥ 70% of relief in pain could clinically predict SR with high accuracy. Platelet count, lower levels of blood glucose and uric acid, distal lesions and LAD as infarct-related artery (IRA) were independently associated with SR. More importantly, SR may result in lower rate of in-hospital events despite less stent placement.

Previous studies [6–8] have suggested that STEMI patients with SR had improved left ventricular function, lower incidence of congestive heart failure and decreased short-term mortality, as compared to patients without SR. In the current study, we also indicated significantly better in-hospital outcome among patients with SR. The possible mechanism could be: 1) SR may be associated with earlier opening time in occluded arteries, resulting in smaller myocardial infarct size. 2) In our study, 23.1% of patients in the NSR group did not achieve blood flow of TIMI grade 3, which may result in poor outcomes. Furthermore, when we only compared patients having TIMI grade 3, SR was still associated with lower in-hospital events. We therefore speculate that the SR group might have more effective reperfusion not only by the reopening of epicardial arteries but also in the microcirculatory level, as compared to patients in the NSR group. 3) Our data demonstrated that stent placement could be safely deferred in the patients with SR. The SR group had a lower rate of stent placement (10.5% vs. 84.6% in emergent procedure and 60.5% vs. 86.4% totally) which might be associated with less procedure-related complications as compared to NSR group. Future studies are needed to confirm our speculations regarding the mechanism of benefit associated with SR.

Although SR was associated with favorable in-hospital events, but no significantly difference was verified with regarding to the long-term follow up. The MACCE were 2.6% in SR group and 6.9% in NSR group, which were relatively lower compared to previous studies [15]. This may somewhat reflect the improvement on management of STEMI and therefore diminish the gap on prognosis between the two groups. Moreover, patient amount was relatively small in the current study, which may impair the power of statistical analysis.

In fibrinolytic era, complete resolution of ST segment elevation, early peaking of biomarkers, reperfusion arrhythmias and relief of chest pain, has been investigated for noninvasive assessment of TIMI grade 3 after thrombolytic therapy, indicating patency of the culprit vessel [16–18]. Even so, clinical features are still considered to be poor markers of reperfusion, without any combinations of them being more able to predictive coronary artery patency more reliably [19]. In our study, when ST segment resolution ≥ 70% and symptom relief ≥ 70% were combined to predict SR, the coincidence rate of patients both with ≥ 70% ST-segment resolution and ≥ 70% relief of chest pain and SR was 100%. Thus, it may be feasible for physicians to accurately identify the restoration of coronary blood flow in patients with STEMI, based on those clinical features.

Since endogenous fibrinolysis or pretreatment with anti-thrombotic agents is regarded to be conducive to SR [2], some clinical characteristics may be associated with SR. Rimar D [10], et al. found the incidence of SR was 4% of STEMI patients, and old age, preinfarction angina, hypertension, non-Q wave myocardial infarction, and Killip class I on admission were independent variables associated with SR. However, Stone GW [2], et al. indicated the rate of SR is 16% but no baseline characteristics clearly predicted SR. In this study, we founded that higher level of uric acid and blood glucose were independently related to SR. Akpek M [20], et al. reported uric acid had a negative relation with TIMI blood flow in STEMI patients undergoing primary PCI. Timmer JR [21], et al. showed hyperglycemia could predict an initial TIMI flow grade 0 to 2 before primary PCI in STEMI patients. Theoretically, uric acid may promote activation of clotting factors, inhibit natural anticoagulant and lead to mural thrombosis through inflammation or platelet pathway, therefore inhibit thrombus autolysis [22–23]. Hyperglycemia could increase activation platelet, damage endothelium-dependent vasodilation and increased inflammatory responses, finally affect platelet function, blood coagulation and fibrinolysis, leading to thrombosis hard to dissolve [24–25]. Interestingly, our data showed patients in SR group had lower platelet levels compared to NSR group. We speculate that these patients may have more severe platelet consumption due to higher thrombotic burden in the culprit lesion. Further studies are needed to elucidate the mechanism.

### Study limitations

Limitations of the study include that all data were collected from one institution which may leave selection bias. Moreover, we could not identify the exact duration between coronary blockage and reopening in SR patients. Also, we did not have data about peak levels of CK-MB or troponin to represent and compare the myocardial damage between the SR and NSR groups. Additionally, we speculated that less PCIs might be associated less procedure-related complications, which may partly account for the favorable outcome in the SR group. However, no consolidate data supported it. Further studies are needed to confirm this point. Finally, this study could not reflect influence of new anti-thrombosis agents on SR.

## CONCLUSIONS

Spontaneous reperfusion is not rare daily practice. SR is associated with favorable outcomes, despite of less stent usage. Stent placement could be safely deferred in the patients with SR.
